# Antiretroviral treatment adherence and its determinants in Sub-Saharan Africa: a prospective study at Yaounde Central Hospital, Cameroon

**DOI:** 10.1186/1742-6405-6-21

**Published:** 2009-10-12

**Authors:** Mathieu Rougemont, Beat E Stoll, Nadia Elia, Peter Ngang

**Affiliations:** 1 Institute of Social and Preventive Medicine, CMU, CH-1211 Geneva 4, Switzerland; 2 Department of Internal Medicine, CNPS Hospital, Yaoundé, Cameroon

## Abstract

**Background:**

With African health-care systems facing exploding demand for HIV care, reliable methods for assessing adherence and its influencing factors are needed to guide effective public-health measures. This study evaluated individual patient characteristics determining antiretroviral treatment (ART) adherence and the predictive values of different measures of adherence on virological treatment failure in a cohort of patients in a routine-care setting in Cameroon.

**Methods:**

Longitudinal study over 6-months following ART introduction, using patients questionnaires and hospital and pharmacy records.

**Results:**

At the end of the 6 months study period, 219 of 312 patients (70%) returned to the pharmacy to refill their medication, 17% (51) were lost to follow-up, 9% (28) were dead and 4% (14) were transferred to other care centres. Virological treatment failure at 6 months was experienced by 26 patients, representing 13% of patients with available viral load value. Pharmacy refill irregularity was the most powerful predictor (odds ratio 12.4; P < 0.001) of virological treatment failure, compared with CD4 cell count increase at 6 months (odds ratio 7.8; P = 0.002) or self-reported adherence at one month (odds ratio 1.1; P = 0.85). Low intensity of ART side-effects after one month was strongly associated with survival (odds ratio 0.11; P = 0.001). Patients starting ART with CD4 cell count <100 cells/mm^3 ^had a greater risk of dying during the follow-up period (odds ratio 2.69; P = 0.02). Compared with asymptomatic CDC stage A patients, CDC stage B (odds ratio 5.72) and CDC stage C patients (odds ratio 16.9) had higher risk of becoming lost to follow-up (P < 0.001). In the multivariate analyses, pharmacy non-adherence was less frequent in women (adjusted odds ratio 0.56; P = 0.05) but more frequent in patients with high monthly income (odds ratio 3.24; P = 0.04).

**Conclusion:**

Pharmacy-refill adherence might be considered as an alternative to CD4 count monitoring for identification of patients at risk of virological failure, especially in resources-scarce countries. The study confirmed the difficulty in demonstrating clear associations of individual patient factors and treatment outcomes. The substantial loss to follow-up and deaths occurring within 6 months after initiating ART emphasise the need to understand the best timing of ART initiation and further elucidate and educate on the underlying reasons for delaying initiation of ART in resource-limited countries

## Background

During the last decade, access to HIV care in Sub-Saharan Africa has been improved by reduction in the cost of ART and by the implementation of WHO guidelines promoting scaling-up by task shifting for clinical decision-making to less specialised health-care workers [1]. However, the challenge to achieve high adherence to ART is particularly acute in Sub-Saharan Africa as the high rates of HIV/AIDS lead to greater absolute numbers of affected individuals than in other low-income regions. Although long-term good ART adherence has been observed in certain settings of public sectors in Africa (Nachega, data presented at 16th Conference on Retroviruses and Opportunistic Infections 2009), the magnitude of this challenge in Sub-Saharan Africa remains large [2] and there is growing evidence for high rates of patients loss to follow-up [3,4]: a recent review reported that ART programmes in Africa retain only about 60% of their patients after two years on ART [5].

As in other African countries, the average prevalence of HIV in Cameroon has risen dramatically during the last two decades, from 0.5% in the early 1990s to 5.5% in 2004 [6]. In view of the severe socio-economic and developmental impact of the epidemic, the government of Cameroon has made the fight against HIV/AIDS a priority area in its 2000-2010 strategic plans to combat poverty. Although the cost of drugs has gone through several phases of reduction since a pilot antiretroviral drug delivery programme started in 2000, the implementation of a national decentralisation programme for HIV care in 2006 led to existing health infrastructures being overwhelmed by a huge demand for treatment. The overcrowding of HIV care centres has recently increased, as free treatment began to be available in May 2007.

Adherence to antiretroviral therapy (ART) is crucial to ensure viral suppression, decrease the risk of disease progression and drug resistance. However, it is difficult to measure accurately, which is reflected in the number of conflicting reports available on the response to ART in people living with HIV/AIDS (PLHIV). Optimistic reports [7-11] may have over-emphasised selective publication of positive results [12], or have been biased towards highly motivated patients with early access to limited therapy [13]. Given these methodological difficulties, it is not surprising that a bewildering number of factors have been reported to influence adherence: age; gender; monthly income; level of education; travel time from home to clinic; baseline CD4 cell count; CDC HIV clinical stage before starting ART, type of ART regimen, presence of early ART side-effects and disclosure of HIV status to at least one relative [14-20].

Clearly, there is a need for simple and reliable methods of assessing adherence as well as its influencing factors in order to guide effective public-health measures.

The objective of this study was to compare the diagnostic accuracy of CD4 cell count changes, self-reported adherence and pharmacy refill history to identify patients with virological treatment failure amongst a cohort of PLHIV in a routine care setting in Cameroon during the first 6-month follow-up period. We then examined the relationship between patient individual factors and adherence, including in our analysis the substantial number of patients loss to follow-up.

## Methods

### Study site, population and design

The Day Hospital of Yaoundé Central Hospital (YCH) opened in 1998 with a capacity of offering care to 4500 PLHIV. By 2006 this centre had registered more than 10,000 PLHIV, with approximately 2000 patients on ART, resulting in long waiting periods, overburdened staff and a severe shortage of clinic space.

At the time of the study, patients had to pay for care, including drugs (USD 7 to 17 monthly), laboratory tests (USD 50 for pre-treatment check-up) and clinical visits (USD 4 for a ticket valid for one month). Before receiving ART, pre-counselling and post-counselling visits were necessary, followed by three consecutive medical visits and a final socioeconomic inquiry to assess ART readiness. Therapeutic committee determined eligibility for ART based on CDC clinical staging (the classification routinely used in this hospital) and CD4 cell counts.

We recruited all ART-naïve patients at the Day Hospital of YCH between June and September 2006 at their first antiretroviral prescription visit. Exclusion criteria were age under 18 years, imminent transfer to another treatment centre, prior antiretroviral therapy and poor health status, with the patient unable to provide consent and respond to the initial inclusion questionnaire. In most cases the latter patients were hospitalised in a common room that rendered confidentiality difficult.

Routine clinical visits where scheduled on day 15, at months 1 and 3, and every 3 months thereafter. Antiretroviral therapy was based on triple-drugs regimens consisting of two NRTIs and one non-NRTI and was dispensed only at the central hospital's pharmacy. Patients were provided with more medication than required, i.e. tablets were usually dispensed for 30 days, whereas pharmacy visits were scheduled in multiples of 4 weeks. At each pharmacy visit were registered: date of present prescription refill, prescribed drug regimen and date of the next pharmacy appointment.

This study was approved by the Cameroon National Ethics Committee and informed written consent was obtained from each participant prior to inclusion into the study.

### Data collection

Biomedical and clinical data were extracted from the clinic file of each enrolled patient and included biochemistry, haematological tests, CD4 cell counts determinations (FacsCount; Becton-Dickinson, San Jose, CA, USA), date of death or transfer to another health centre. Determination of CD4 cell levels were performed at the pre-treatment examination and routinely requested every 6 months thereafter. Plasma viral load assays (Abbott Real Time HIV-1 assay) were offered to all study participants after ≥ 5 months of ART.

At the day of introducing ART, three trained study interviewers administered an initial questionnaire exploring socioeconomic status, knowledge and beliefs toward HIV and ART, social support and disclosure of HIV status [21,22].

A second culturally adapted questionnaire gathered information on self-reported ART adherence and early ART side-effects [23]. It was administered after one month of therapy by a PLHIV external to the health care team. We asked whether or not any ART had been missed, using global one-month recall. All interviews were conducted in either French or English, the two national languages in Cameroon.

Pharmacy records were reviewed 6 months after ART initiation to define pharmacy-refill adherence. In order to further ascertain true adherence status, patients without renewed prescriptions in the last two months were actively traced by two phone calls and if still alive, encouraged to come back to the clinic to restart therapy.

### Outcome definitions

Virological treatment failure was defined as a viral load >400 copies/ml (lower detection limit of 40 copies/ml), and was used as the gold standard to compare different methods of adherence measurements: CD4 count change, self reported adherence and pharmacy-refill history. Immunological treatment failure was defined as a reduction in CD4 count after 6-months of treatment to, or below, pre-therapy baseline, or persistent levels below 100 cells/mm^3 ^[1].

Self-reported adherence was classified as "adherent" when not a single dose was missed or non-adherent if the patient admitted having missed at least one dose during the last month.

Pharmacy non-adherence was defined as renewal of prescriptions of later than 2 weeks after the scheduled pharmacy appointment, or patients confirming abandoned ART on phone call tracing. Patients not traced by phone calls were considered to be lost to follow-up. The follow-up time was censored at the last scheduled pharmacy visit.

### Statistical Analysis

Statistical analyses were conducted using STATA version 9 (Texas, USA).

The three different methods to measure adherence were compared in their ability to predict virological failure by computing their sensitivity and specificity, positive and negative predictive values and positive and negative likelihood ratios. Finally, the association between each method and virological failure was also reported as OR and 95% confident interval. We then used the method of adherence assessment showing the strongest association with virological failure as a surrogate of true treatment adherence. Chi-square tests were used to determine the associations between each individual patient baseline characteristic and adherence, at a P < 0.2 level of significance. Logistic regression models were fitted including characteristics shown to be associated with adherence in univariate analysis (P < 0.2). Variables measured at one month were excluded from this analysis as the substantial number of patients with early loss to follow-up reduced the number of available data and thus statistical power.

**Figure 1:**
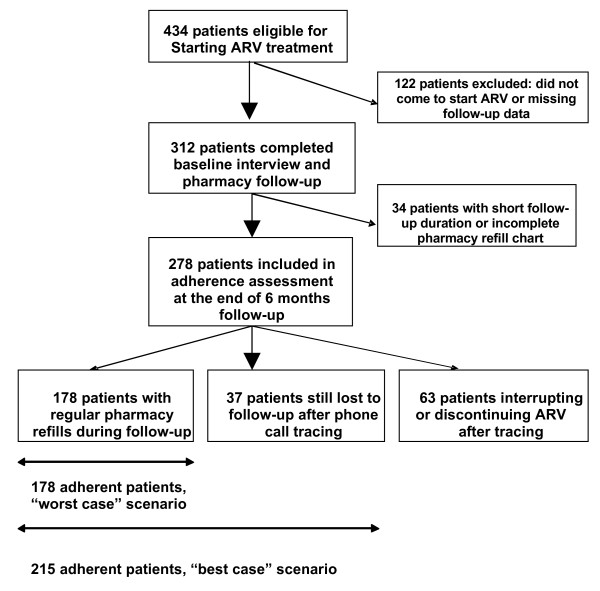
**Flow chart for adherence analysis**.

As the substantial rate of patients loss to follow-up may introduce bias into estimates of risk factors for treatment non-adherence, we conducted a sensitivity analysis where associations were tested in two scenarios: a best-case scenario, where all patients lost to follow-up and not successfully traced by phone call were considered as adherent, and a worst-case scenario where all such patients were defined as non-adherent [Fig. 1].

## Results

### Baseline characteristics

Patient's disposition is shown in [Fig 1]. Of the 434 patients selected to receive ART between June and September 2006, 405 (93%) attended the baseline visit and 312 (72%) were included in the study. Reasons for non participation of eligible patients were imminent transfer to another treatment centre (n = 20), first antiretroviral prescription by a physician not participating in the study (n = 15), a physical health status rendering long interviews difficult for the patient (n = 11), refusal to take part (n = 8) and other reasons (n = 39).

Among the 312 enrolled patients at the start of ART, the mean age (± SD) was 37 ± 9 years, with a majority of women (63%) [Table 1]. Although 97 participants (31%) reported no income-generating occupation, 76 (78%) of these received occasional or regular financial help from relatives. Thirty-two patients (10%) benefited from treatment support from the Global Fund, which was associated with neither socio-economic status nor with income levels (data not shown). Forty-six per cent of the cohort had the lowest financial income (monthly income of less than USD50). About two-thirds of the patients (70%) lived close to the clinic, with only 15 (5%) living more than 4 hours from the Day Hospital by public transport.

**Table 1:** Socio-demographic and clinical data at start of ART of the 312 patients studied

**Characteristics**	**n**	**%**
**Age groups (year)**		
<30	65	21
30-49	199	64
≥ 50	32	10
Missing data	16	5

**Gender**		
Male	106	34
Female	198	63
Missing data	8	3

**Marital status**		
Single, Divorced, Separated	182	58
Married, Cohabiting	114	37
Missing data	16	5

**Level of education**		
Primary	91	29
Secondary without Bachelor	156	50
Secondary with Bachelor or University	45	15
Missing data	20	6

**Monthly income**		
< USD 50	145	46
USD50 - USD 125	73	23
> USD 125	77	25
Missing data	17	6

**Travel time (home to clinic) (h)**		
<1	217	70
1-4	68	21
>4	15	5
Missing data	12	4

**Time of follow-up before ART**		
<1 month	195	63
1-6 months	86	27
>6 months	20	6
Missing data	11	4

**CDC Stage**		
A	57	18
B	201	64
C	44	14
Missing data	10	4

**CD4 baseline Strata (cells/mm^3^)**		
<100	143	46
100-200	109	35
>200	47	15
Missing data	13	4

Most patients (n = 195 or 63%) had been followed at the clinic for less than one month before initiating ART. However, only 36 patients (12%) reported the use of alternative therapies for HIV in the past. At the time of starting ART the majority of the cohort were classified as symptomatic (64% at CDC stage B and 14% at stage C), the median baseline CD4 count was 104 cells/mm^3 ^(interquartile range 50 - 177 cells/mm^3^). All patients received two nucleoside reverse transcriptase inhibitors (NRTIs) and one non-nucleoside reverse transcriptase inhibitor (NNRTI) with the exception of one patient who received two NRTIs and one protease inhibitor.

When asked about social support and stigmatisation, 275 participants (88%) reported having disclosed their HIV status to at least one person and 260 (83%) declared satisfied by family or friends support. Most patients (n = 258 or 80%) had optimistic expectations of the effects of ART on their health status, a sizable minority believed that treatment could cure HIV (44%) and 118 patients (38%) were unaware of possible ART side effects.

### Treatment outcomes and adherence measures

Of 312 patients in the initial cohort, 219 (70%) were still coming to the pharmacy to refill their medication after 6 months of follow-up, while 51 (17%) were lost, 28 (9%) had died and 14 (4%) were had been transferred to other care centres. The incidence rate of loss to follow-up was 40.1 per 100 persons years (95% CI: 30.5 - 52.8) and mortality rate was 21.2 per 100 persons years (95% CI: 15 - 31). Drop-out occurred at a median of 44 days (IQR: 28 - 80 days) and death occurred at a median of 55 days (IQR: 28 - 81 days) after treatment initiation.

The first routinely scheduled follow-up CD4 cell count was performed by only 71 patients (23% of patients still followed-up). The median rise in CD4 cell count from baseline to this point was 117 cells/mm^3 ^and 16 patients (22%) with available follow-up CD4 cell count met criteria for immunological treatment failure. Using phone call tracing alongside tracking of patients during regular follow-up visits, we obtained viral load values for 206 patients. Virological treatment failure (viral load value of > 400 copies/ml) at 6 months was experienced by 26 patients, representing 13% of patients with available viral load value and 8% of the entire cohort.

Self-reported adherence data after one month of therapy were obtained for 238 patients (76%). The majority of respondents (78%) claimed not to have missed a single dose during this period. The proportion of participants that reported full adherence to treatment during the past month decreased from 83% at one month to 57% at 6 months.

Adherence based on pharmacy refill charts after 6 months of follow-up could be analysed for 278 participants. Twenty-eight subjects were excluded because of their short follow-up duration (<2 months) and 6 patients had incomplete data on the refill chart [Fig 1]. Two-thirds (64%) of patients assessed came regularly every month within two weeks of the pharmacy-appointed dates and qualified as adherent in the analysis. Non-adherence (interruptions or discontinuations) was defined for 23% of the participants with available data: 49 subjects interrupted their treatment for at least 3 weeks during the follow-up period; 14 patients lost to follow-up could be traced by phone calls and confirmed they had discontinued ART. Thirty-seven patients (14%) could not be traced by phone call after loss from pharmacy follow-up.

### Adherence measurements and virological treatment failure

There were significant differences between the ability of different measures of adherence to predict virological treatment failure after 6 months of therapy [Table 2]. Pharmacy-refill irregularity was the most powerful predictor (OR, 12.40; 95% CI, 4.75-32.40; P < 0.001). In the sub-sample of 194 patients whose 6-month viral load and pharmacy refill charts were available, 38% of the pharmacy non-adherent patients demonstrated virological treatment failure while 95% of pharmacy adherent patients presented with virological suppression. Immunological treatment failure was also significantly associated with virological treatment failure (OR, 7.78; 95% CI, 1.68-36.0; P = 0.002). However, pharmacy adherence estimated by pharmacy refill charts had greater accuracy for detecting virological treatment failure than CD4 count changes at 6-months: the sensitivity was higher (72% versus 53%) with approximately the same specificity (82% versus 88%).

**Table 2:** Potential predictors associated with virological treatment failure (>400 HIV RNA cop/ml) after 6 months of initiating ART

	**Self reported adherence**		**CD4 count change**		**Pharmacy refill history**	
	
	**100%**	**<100%**	**Immunological success**	**Immunological failure**	**Regular, continuous**	**Irregular or interrupted**
**Total (Nb)**	140	25	51	14	147	47

**Virological success (Nb)**	125	22	44	6	140	29

**Virological failure (Nb)**	15	3	7	8	7	18

**OR (95%CI)**	1.13 (0.30-4.25)		7.78 (1.68-36.0)		12.4 (4.75-32.4)	

**Sensitivity**	0.17		0.53		0.72	
**Specificity**	0.85		0.88		0.83	

**Positive predictive value**	0.12		0.57		0.38	
**Negative predictive value**	0.83		0.86		0.95	

**Positive likelihood ratio**	1.13		1.23		4.23	
**Negative likelihood ratio**	0.97		0.53		0.34	

Viral load measurement was available for 206 patients. Immunological success means a rise above 100 cells/μl; immunological failure means a drop to pre-treatment levels or <100 cells/μl,)

Notably, self-reported adherence at 1 month was not significantly associated with virological treatment outcomes.

### Associations between patient characteristics, retention and mortality

Univariate analysis of individual patient factors [Table 3] showed that the most significant factor associated with survival was the improvement of early ART side-effects, (OR: 0.11; 95% CI: 0.02-0.52). Patients starting treatment with CD4 cell count below 100 cells/mm^3 ^were at significantly greater risk of death during the follow-up period (OR: 2.69; 95% CI: 1.12-6.44). We found only a trend for association of HIV CDC stage with mortality. However, HIV CDC clinical stage at the beginning of treatment significantly predicted (P < 0.001) loss to follow-up: compared with asymptomatic patients CDC stage A, CDC stage B patients (OR: 5.72; 95% CI: 1.33-24.70) and specially CDC stage C patients (OR: 16.90; 95% CI: 3.58-80.30) had greater rates of loss to follow-up.

**Table 3:** Logistic regression of patient characteristics associated with treatment outcomes in the first 6 months after initiating ART

**Characteristics**	**Disappeared (n = 51)**		**Died (n = 28)**	
	
	**Odds ratio** **(95%CI)**	**P-value**	**Odds ratio** **(95%CI)**	**P-value**
**Age (years)**		0.65		0.23
<30	0.98 (0.45-2.15)		0.39 (0.11-1.35)	
30-49	1		1	
≥ 50	1.55 (0.61-3.96)		0.58 (0.13-2.65)	

**Sex**		**0.09**		0.37
Male	1		1	
Female	0.58 (0.31-1.08)		0.68 (0.30-1.55)	

**Monthly income (USD)**		0.9		0.43
<USD50	1.19 (0.55-2.59)		0.92 (0.32-2.63)	
USD50 - USD125	1		1	
>USD125	1.09 (0.44-2.68)		1.67 (0.57-4.90)	

**Education**		0.44		0.51
Primary	1		1	
Secondary without bachelor	1.38 (0.67-2.84)		1.71 (0.64-4.58)	
Secondary with bachelor or university	0.79 (0.26-2.39)		1.77 (0.49-6.01)	

**Travel time to clinic (hour)**		0.47		0.97
<1	1		1	
1-4	1.30 (0.62-2.72)		1.02 (0.39-2.71)	
>4	1.93 (0.65-5.75)		1.22 (0.26-5.77)	

**Baseline CD4-cell count (cells/μl)**		0.86		**0.02**
<100	1.06 (0.57-1.97)		**2.69 (1.12-6.44)**	
≥ 100	1		1	

**Clinical stage**		**<0.001**		0.11
CDC stage A	1		1	
CDC stage B	**5.72 (1.33-24.70)**		2.08 (0.59-7.35)	
CDC stage C	**16.9 (3.58-80.30)**		4.52 (1.04-19.70)	

**ART side-effects at 1 month**		0.71		0.638
No side-effects	1		1	
One or more side-effects	1.24 (0.40-3.85)		1.43 (0.30-6.73)	

**ART side-effects at 1 month**		0.14		**0.001**
Improving	0.49 (0.19-1.26)		**0.11 (0.02-0.52)**	
Not improving	1		1	

**Disclosure of HIV status**		0.22		0.93
Yes	1		1	
No	1.10 (0.95-1.27)		1.01 (0.79-1.29)	

Patients who disappeared (n = 51) or died (n = 28) are compared with patients with complete follow-up or transferred (n = 233). OR were calculated using logistic regression, p-values from the likelihood ratio test.

There was a non-significant trend to lower rates of interruption of follow-up for women (OR: 0.58; 95% CI: 0.31-1.08) and for those reporting improving ART side-effects after one month of treatment (OR: 0.49; 95% CI: 0.19-1.26). None of the socio-economic determinants studied appeared to influence patient retention and mortality: neither age, nor level of education, nor economic situation and social support were significantly associated with loss to follow-up or with death.

### Associations between patient characteristics and pharmacy adherence

As patients lost to follow-up may have introduced bias into the analysis, we analysed the association of individual patient characteristics with pharmacy adherence in a sensitivity analysis, using two scenarios, a best-case and a worst-case scenario [Table 4]. In the best-case scenario, where all participants lost to follow-up were analysed as adherent, greater age (OR: 0.38; 95% CI: 0.11-1.33), female sex (OR: 0.66; 95% CI: 0.37-1.18) and patients reporting improvement of early ART side-effects (OR: 0.51; 95% CI: 0.23-1.10) tended keep pharmacy appointments less irregularly (P < 0.2). In this scenario, a monthly middle income was significantly associated (P = 0.01) with greater pharmacy adherence. Low (OR: 2.49; 95% CI: 1.03-6.01) or high (OR: 3.76; 95% CI: 9.58) incomes groups showed a higher risk for pharmacy non-adherence. In the worst-case scenario, female sex (OR: 0.60; 95% CI: 0.35-1.01) and improving early ART side-effects (OR: 0.47; 95% CI: 0.24-0.95) were characteristics associated (P < 0.05) with lower pharmacy non-adherence. CDC stage B patients (OR: 2.68; 95% CI: 1.26-5.68) and specially CDC stage C patients (OR: 5.13; 95% CI: 2.02-13.00) had higher risk of pharmacy non-adherence than asymptomatic patients.

**Table 4:** Patient characteristics associated with pharmacy non-adherence during the first 6 months after initiating ART

**Characteristics**	**Best-case scenario**		**Worst-case scenario**	
	
	**OR (95%CI)**	**P-value**	**OR (95%CI)**	**P-value**
**Age (years)**		**0.09**		0.95
<30	1.45 (0.74-2.83)		1.05 (0.57-1.95)	
30-49	1		1	
≥ 50	0.38 (0.11-1.33)		0.90 (0.40-2.04)	

**Sex**		0.17		**0.05**
Male	1		1	
Female	0.66 (0.37-1.18)		0.60 (0.35-1.01)	

**Monthly income (US$)**		**0.01**		**0.08**
< USD 50 (20'000 FCFA)	2.49 (1.03-6.01)		1.73 (0.89-3.38)	
USD 50 - USD125 (20'000-50'000)	1		1	
> USD 125 (>50'000)	3.76 (1.47-9.58)		2.27 (1.08-4.77)	

**Education**		0.82		0.16
Primary	1		1	
Secondary without Bachelor	0.96 (0.51-1.83)		1.35 (0.76-2.37)	
Secondary with Bachelor or University	0.74 (0.28-1.95)		0.64 (0.27-1.54)	

**Travel time to clinic (hour)**		0.21		0.73
<1	1		1	
1-4	0.88 (0.44-1.78)		1.24 (0.68-2.61)	
>4	0.22 (0.03-1.74)		1.29 (0.44-3.79)	

**Baseline CD4-cell count (cells/μl)**		0.75		0.85
<100	1.09 (0.62-1.93)		1.05 (0.64-1.73)	
≥ 100	1		1	

**Clinical stage**		0.44		**0.001**
CDC stage A	1		1	
CDC stage B	1.60 (0.72-3.55)		2.68 (1.26-5.68)	
CDC stage C	1.72 (0.62-4.75)		5.13 (2.02-13.0)	

**Initial ART regimen**				
Twice daily	1	0.81	1	0.79
Three times daily	0.93 (0.53-1.64)		1.07 (0.65-1.75)	

**Occurrence of ART side-effects at 1 month**		0.46		0.64
No side-effects	1		1	
One or more side-effects	0.74 (0.34-1.62)		0.84 (0.41-1.73)	

**Course of ART side-effects at 1 month**		**0.09**		**0.04**
Improving	0.51 (0.23-1.10)		0.47 (0.24-0.95)	
Not improving	1		1	

**Reported ART adherence at 1 month**				
100%	1	0.55	1	0.19
<100%	1.30 (0.56-3.01)		1.66 (0.78-3.52)	

**Disclosure of HIV status**		0.82		0.38
Yes	1		1	
No	0.98 (0.81-1.18)		1.07 (0.92-1.24)	

Non-adherent patients are compared with adherent patients in a best and a worst case scenario (best-case scenario: patients lost to follow-up were considered adherent; worst-case scenario: patients lost to follow-up were considered non-adherent). The associations of each characteristic with time stayed adherent were examined using logistic regression models.

In the multivariate analysis [Table 5], women presented lower risk for non-adherence both in the best case (adjusted OR: 0.56; 95% CI: 0.29-1.07; P = 0.08) and in the worst case scenarios (adjusted OR: 0.56; 95% CI: 0.31-1.02; P = 0.05). Economic status, in particular patients with the highest monthly income when compared with monthly middle income (OR: 3.24; 95% CI: 1.24-8.46; P = 0.04), was retained as a predictor of poor adherence only in the best case scenario. When compared with asymptomatic patients, the multivariate analysis confirmed a marked risk of non-adherence for CDC stage B patients (OR: 2.75; 95% CI: 1.23-6.18) and CDC stage C patients (OR: 5.07; 95% CI: 1.87-13.80) in the worst-case scenario (P = 0.003).

**Table 5:** Multivariate logistic regression of baseline characteristics associated (P < 0.2) with pharmacy non-adherence in the univariate analysis

**Characteristics**	**Best-case scenario**		**Worst-case scenario**	
	
	**Adjusted OR** **(95%CI)**	**P-value**	**Adjusted OR** **(95%CI)**	**P-value**
**Sex**		**0.08**		**0.05**
Male	1		1	
Female	0.56 (0.29-1.07)		0.56 (0.31-1.02)	

**Monthly income (USD)**		**0.04**		0.28
<USD50	2.04 (0.82-5.06)		1.66 (0.80-3.44)	
USD50 - USD125	1		1	
> USD125	3.24 (1.24-8.46)		1.86 (0.82-4.22)	

**Clinical stage**				**0.003**
CDC stage A			1	
CDC stage B			2.75 (1.23-6.18)	
CDC stage C			5.07 (1.86-13.8)	

## Discussion

Despite substantial improvements in the affordability and availability of ART in recent years, African health-care systems face enormous challenges in the context of exploding demand for HIV care [24]. The main objective of this study was to identify factors influencing ART adherence and evaluate related outcome of therapy 6 months after ART initiation in a routine setting.

### Treatment outcomes and adherence measurements

Our cohort showed a relative low patient retention rate: 70% of the patients who started ART still came to the pharmacy to take their prescription after six months of follow-up, 17% disappeared, 9% died and 4% were referred to other care centres. These results confirm that most losses to follow-up and deaths occur during the initial period after ART initiation.

A recent systematic review [5] of 33 earlier cohorts in developing countries reported a mean retention rate of 79% at six months. Our slightly lower retention rates may be due to the increasing challenge of managing growing numbers of patients treated.

Pharmacy-refill history gives no description of daily adherence to treatment, because patients may not take all prescribed medications. It could also be considered as a time-consuming monitoring tool for the pharmacy staff, owing to the rapidly growing number of patients in public ART programs. However, this is a simple, inexpensive approach and it was previously reported to be as accurate as CD4 counts for predicting virological response [25]. We found the same correlation between pharmacy-refill adherence and virological outcome at 6 months. In our settings, pharmacy-refill adherence had even greater accuracy, with higher sensitivity and similar specificity to CD4 count changes at 6 months for predicting virological treatment failure. Moreover, data from refill charts in routine-care conditions were available for 95% of the participants, whereas only 23% of the cohort performed their first CD4 cell count follow-up, an analysis that patients had to cover at their own expense. The ability of adherence monitoring from the pharmacy to identify patients at risk of treatment failure may help health-care providers for early adherence counselling interventions. At the time of the study, data from refill charts were kept at the Central Pharmacy and were not communicated to clinicians. A practical implication of our findings is that systematic monitoring of pharmacy-refill adherence should be integrated into therapeutic score cards carried by every patient.

Self-reported adherence has been described in several studies as a rapid and inexpensive method, albeit subject to social-desirability and recall biases [26,27]. In our cohort, self-reported adherence was not predictive of virological treatment failure. One earlier study in a South African cohort [28], found only a modest increase in risk (unadjusted OR: 2.35, 95% CI: 1.52-2.53) for patients reporting <100% at 6 weeks for virological failure at 12 months, which might not have been detectable in our smaller population. In addition, the proportion of our participants that reported 100% adherence to treatment during the past month decreased from 83% at one month to 57% at 6 months. Mannheimer [27] described a similar decline in self-reported adherence with time. It is likely that these decreases reflect decreases in true daily adherence with time. However, there may also have been an overestimation of self-reported medication in early stages due to desire to please when patients do not feel confident in a new environment. Further studies in lower-income settings are needed to verify the accuracy of self-reported medication adherence as a predictor of virological outcome.

### Determinants of ART adherence

We examined determinants of adherence in two scenarios to reduce bias due to loss to follow-up. We identified female sex, middle monthly income and less ART related side-effects at one month as predictors of higher pharmacy adherence under both scenarios. Age and HIV clinical CDC stage correlated with pharmacy adherence only under best and worst-case scenarios, respectively. Gender remained of borderline significance after adjusting for potential confounders in the multivariate model.

To the best of our knowledge, the current study is the first to demonstrate that income may not be linearly associated with adherence: patients with monthly middle income had greater pharmacy adherence rates than both the poorest and the richest participants. A recently published meta-analysis [17] examined the association between socio-economic status and adherence to antiretroviral therapy: out of 8 studies, only 2 prospective studies identified low income as a predictor of non-adherence. All, except one [29], analysed income as a binary variable which could explain why none of them described our U-shaped association. Selection bias through restricted financial access to health care seems unlikely in our settings: two-thirds (67%) of patients reported no or occasional income, whereas only 25% of our population reported earning more than USD125 per month.

With the exception of a trend towards greater loss rates among men, we failed to demonstrate any other social or demographic association with loss to follow-up. The same gender association with both death and loss to follow-up was recently reported from a study in Malawi [30].

### Limitations of the study areas of uncertainty

It is unclear how far our results can be generalised to other countries and healthcare systems. Compared with reports from patients starting ART in other treatment programmes in lower-income countries [2], our cohort had a lower proportion of men, fewer patients at clinical advanced disease stages and higher baseline CD4 cell counts.

The percentage of male patients in our cohort reflects the gender distribution of HIV prevalence in Cameroon, which indicates that women's access to health care for HIV is improving. We also observed discrepancies between biological (CD4 counts) and clinical (CDC stages) levels of disease in our participants: Only 14% of our patients started therapy at CDC clinical stage C, despite a median CD4 cell count of 107 cells/μl. The simplest explanation for this is an underestimation of CD4 cell count at the Day Hospital Laboratory. This conclusion is supported by our observation of a large variability of baseline CD4 cell counts for patients who were analysed at close intervals at different laboratories (data not shown). A lack of association between immunological and virological outcomes have been found in similar settings [3] suggesting that CD4 cell count follow-up should be interpreted with caution, particularly if performed in different laboratories. An alternative explanation would be a systematic clinical misclassification of patients. This is supported by the observation that 75 of 169 patients (44%) enrolled at the YCH Day hospital between 2001 and 2003 were classified as CDC clinical stage C [31]. Such inconsistencies also reflect the constraints on hospital resources and pressure on staff generated by the rapidly increasing number of eligible cases to be evaluated by the Therapeutic Committee every week.

Only 15 patients in our study (5%) lived more than 4 hours of travel from the Day Hospital, which may not be representative of patients in ART programmes at other hospitals. A survey of patients initiating ART from 2002 to 2005 in Limbe Provincial Hospital, the only ART clinic serving the Southwest Province of Cameroon at the time of the study, showed that 44.3% of patients were living more than 40 km by inaccessible road from the clinic [19]. As treatment scale-up programmes are currently attempting to shift ART delivery to health districts in remote areas, more research is needed on geographic access to ART.

Only 312 out of 440 originally eligible patients were included in our study cohort. Such high attrition rates before initiating ART treatment are a main source of selection bias in studies of retention rates in Africa. Data from different cohorts in other lower-income settings [2] suggest that about 50% of patients lost early to follow-up may have died. This phenomenon needs to be better understood to enable targeted interventions. Without an informed consent, we were not allowed to review all medical records in detail to assess if baseline characteristics of the initial eligible population differed from our cohort.

Our study focused on the first six months of follow-up after ART initiation, a period previously characterized by high attrition [5,18,19,32] and thus considered to be a crucial phase for promoting adherence. As level of adherence and its predictors may vary over time, we strongly encourage ART programs to conduct long-term surveillance of these outcomes to fully understand the subtle variations of its dynamic behavioral process.

## Conclusion

Although there is no 'gold standard' for the assessment of medication adherence, pharmacy-refill adherence or other easily accessible methods should be considered as an alternative to CD4 count monitoring for identification of patients at risk of virological failure, especially in low-income countries. It represents a simple, inexpensive and accurate method that correlates with virological response to treatment. Data from pharmacy refill charts should be made available to health-care workers to help identifying patients at greatest risk of treatment failure.

It is still difficult to pinpoint determinants of non-adherence to ART in lower-income countries; for example, our study indicates than the role of economic status is more complicated than may previously have been thought. Preventing treatment discontinuation by enhancing adherence counselling for a higher-risk population may not be effective: all previous studies failed to clearly demonstrate a specific group that would benefit from such intervention. Developing strategies should rather focus on improving adherence follow-up by simple and inexpensive measurement.

Finally, more studies in resource-limited countries are urgently needed to understand the underlying reasons for late initiation of ART and for high attrition rates before initiating ART, which account for a large number of early losses to follow-up and deaths in lower-income countries.

## Abbreviations

AIDS: Acquired immune deficiency syndrome; ART: Anti retroviral treatment; CDC: Centers for disease control and prevention; CI: Confidence interval; HIV: Human immune deficiency virus; IQR: Inter quartile range; NNRTI: Non- Nucleoside reverse transcriptase inhibitor; NRTI: Nucleoside reverse transcriptase inhibitor; OR: Odds ratio; P: P value for statistical significance; PLHIV: People living with human immune deficiency virus; SD: Standard deviation; WHO: World Health Organization; YCH: Yaoundé Central Hospital.

## Competing interests

The authors declare that they have no competing interests.

## Authors' contributions

All authors read and approved the final manuscript. MR conceived the study, raised funds, participated in the design and coordination of the study, collected and cleaned data, interpreted data, drafted the manuscript. BES contributed to the design and coordination of the study, contributed to data entering and cleaning, participated to interpreted and helped in drafting and revising the manuscript. NE contributed to data cleaning, performed statistic analysis and interpretation, and helped in revising the manuscript. PN conceived the study, participated in the design and coordination of the study, carried out data collection, coordinated laboratory sample processing, contributed to manuscript revision. All authors read and approved the final manuscript.

## References

[B1] Gilks CF, Crowley S, Ekpini R, Gove S, Perriens J, Souteyrand Y (2006). The WHO public-health approach to antiretroviral treatment against HIV in resource-limited settings. Lancet.

[B2] Brinkhof MW, Dabis F, Myer L, Bangsberg DR, Boulle A, Nash D, Schechter M, Laurent C (2008). Early loss of HIV-infected patients on potent antiretroviral therapy programmes in lower-income countries. Bull World Health Organ.

[B3] Van Oosterhout JJ, Bodasing N, Kumwenda JJ, Nyirenda C, Mallewa J, Cleary PR (2005). Evaluation of antiretroviral therapy results in a resource-poor setting in Blantyre, Malawi. Trop Med Int Health.

[B4] Laurent C, Meilo H, Guiard-Schmid JB, Mapoure Y, Noel JM, M'Bangue M (2005). Antiretroviral therapy in public and private routine health care clinics in Cameroon: lessons from the Douala antiretroviral (DARVIR) initiative. Clin Infect Dis.

[B5] Rosen S, Fox MP, Gill CJ (2007). Patient retention in antiretroviral therapy programs in sub-Saharan Africa: a systematic review. PLoS Med.

[B6] (2009). Demographic and Health Surveys Cameroon 2004. http://www.measuredhs.com/countries/metadata.cfm?surv_id=232&ctry_id=4&SrvyTp=ctry.

[B7] Laniece I, Ciss M, Desclaux A, Diop K, Mbodj F, Ndiaye B (2003). Adherence to HAART and its principal determinants in a cohort of Senegalese adults. AIDS.

[B8] Orrell C, Bangsberg DR, Badri M, Wood R (2003). Adherence is not a barrier to successful antiretroviral therapy in South Africa. AIDS.

[B9] Weiser S, Wolfe W, Bangsberg D, Thior I, Gilbert P, Makhema J (2003). Barriers to antiretroviral adherence for patients living with HIV infection and AIDS in Botswana. J Acquir Immune Defic Syndr.

[B10] Laurent C, Kouanfack C, Koulla-Shiro S, Nkoue N, Bourgeois A, Calmy A (2004). Effectiveness and safety of a generic fixed-dose combination of nevirapine, stavudine, and lamivudine in HIV-1-infected adults in Cameroon: open-label multicentre trial. Lancet.

[B11] Attaran A (2007). Adherence to HAART: Africans take medicines more faithfully than North Americans. PLoS Med.

[B12] Weidle PJ, Wamai N, Solberg P, Liechty C, Sendagala S, Were W (2006). Adherence to antiretroviral therapy in a home-based AIDS care programme in rural Uganda. Lancet.

[B13] Mills EJ, Nachega JB, Buchan I, Orbinski J, Attaran A, Singh S (2006). Adherence to antiretroviral therapy in sub-Saharan Africa and North America: a meta-analysis. JAMA.

[B14] Mills EJ, Nachega JB, Bangsberg DR, Singh S, Rachlis B, Wu P (2006). Adherence to HAART: a systematic review of developed and developing nation patient-reported barriers and facilitators. PLoS Med.

[B15] Amberbir A, Woldemichael K, Getachew S, Girma B, Deribe K (2008). Predictors of adherence to antiretroviral therapy among HIV-infected persons: a prospective study in Southwest Ethiopia. BMC Public Health.

[B16] Etard JF, Laniece I, Fall MB, Cilote V, Blazejewski L, Diop K (2007). A 84-month follow up of adherence to HAART in a cohort of adult Senegalese patients. Trop Med Int Health.

[B17] Falagas ME, Zarkadoulia EA, Pliatsika PA, Panos G (2008). Socioeconomic status (SES) as a determinant of adherence to treatment in HIV infected patients: a systematic review of the literature. Retrovirology.

[B18] Karcher H, Omondi A, Odera J, Kunz A, Harms G (2007). Risk factors for treatment denial and loss to follow-up in an antiretroviral treatment cohort in Kenya. Trop MedInt Health.

[B19] Mosoko JJ, Akam W, Weidle PJ, Brooks J, Aweh A Survival and adherence to ART in an era of decreasing drug cost in Limbe, Cameroon. 14th Conference on Retroviruses and Opportunistic Infections (CROI); 2007 25-28 February; Los Angeles, California.

[B20] Ollivier F, N'Kam M, Midoungue C, Rey JL (2005). Study conducted at the Yaounde University Hospital on anti-retroviral treatment compliance (Cameroon). Sante Publique.

[B21] Nachega JB, Lehman DA, Hlatshwayo D, Mothopeng R, Chaisson RE, Karstaedt AS (2005). HIV/AIDS and antiretroviral treatment knowledge, attitudes, beliefs, and practices in HIV-infected adults in Soweto, South Africa. J Acquir Immune Defic Syndr.

[B22] Weiser S, Wolfe W, Bangsberg D, Thior I, Gilbert P, Makhema J (2003). Barriers to antiretroviral adherence for patients living with HIV infection and AIDS in Botswana. J Acquir Immune Defic Syndr.

[B23] Chesney MA, Ickovics JR, Chambers DB, Gifford AL, Neidig J, Zwickl B (2000). Self-reported adherence to antiretroviral medications among participants in HIV clinical trials: the AACTG adherence instruments. Patient Care Committee & Adherence Working Group of the Outcomes Committee of the Adult AIDS Clinical Trials Group (AACTG). AIDS Care.

[B24] Wester CW, Bussmann H, Avalos A, Ndwapi N, Gaolathe T, Cardiello P (2005). Establishment of a public antiretroviral treatment clinic for adults in urban Botswana: lessons learned. Clin Infect Dis.

[B25] Bisson GP, Gross R, Bellamy S, Chittams J, Hislop M, Regensberg L (2008). Pharmacy refill adherence compared with CD4 count changes for monitoring HIV-infected adults on antiretroviral therapy. PLoS Med.

[B26] Chesney MA, Ickovics JR, Chambers DB, Gifford AL, Neidig J, Zwickl B (2000). Self-reported adherence to antiretroviral medications among participants in HIV clinical trials: the AACTG adherence instruments. Patient Care Committee & Adherence Working Group of the Outcomes Committee of the Adult AIDS Clinical Trials Group (AACTG). AIDS Care.

[B27] Mannheimer S, Friedland G, Matts J, Child C, Chesney M (2002). The consistency of adherence to antiretroviral therapy predicts biologic outcomes for human immunodeficiency virus-infected persons in clinical trials. Clin Infect Dis.

[B28] Fielding KL, Charalambous S, Stenson AL, Pemba LF, Martin DJ, Wood R, Churchyard GJ, Grant AD (2008). Risk factors for poor virological outcome at 12 months in a workplace-based antiretroviral therapy programme in South Africa: a cohort study. BMC Infect Dis.

[B29] Singh N, Berman SM, Swindells S, Justis JC, Mohr JA, Squier C, Wagener MM (1999). Adherence of human immunodeficiency virus-infected patients to antiretroviral therapy. Clin Infect Dis.

[B30] Chen SC, Yu JK, Harries AD, Bong CN, Kolola-Dzimadzi R, Tok TS, King CC, Wang JD (2008). Increased mortality of male adults with AIDS related to poor compliance to antiretroviral therapy in Malawi. Trop Med Int Health.

[B31] Laurent C, Bourgeois A, Mpoudi-Ngole E, Ciaffi L, Kouanfack C, Mougnutou R (2008). Tolerability and effectiveness of first-line regimens combining nevirapine and lamivudine plus zidovudine or stavudine in Cameroon. AIDS Res Hum Retroviruses.

[B32] Lawn SD, Myer L, Harling G, Orrell C, Bekker LG, Wood R (2006). Determinants of mortality and nondeath losses from an antiretroviral treatment service in South Africa: implications for program evaluation. Clin Infect Dis.

